# Why a D2 gastrectomy plus adjuvant chemotherapy is insufficient in locally advanced gastric cancer

**DOI:** 10.3332/ecancer.2016.706

**Published:** 2016-12-21

**Authors:** Z Sebastián Solé, Francisco E Larsen, Claudio V Solé

**Affiliations:** IRAM Clinic, Universidad Diego Portales, Americo Vespucio Norte 1314, Chile 7630370

**Keywords:** gastric, cancer, stomach, adjuvant, radiotherapy, chemotherapy, gastrectomy

## Abstract

This review discusses all the important published evidence regarding adjuvant treatments in locally advanced gastric cancer. In this process it revealed facts that demonstrate the superiority of radiotherapy and concomitant chemotherapy to chemotherapy alone. Some outstanding work that has not yet been published is also discussed.

## Introduction

Surgery was for years the exclusive treatment for gastric cancer, but its results are poor. In the best series, the survival rate is around 30–40% when there are advanced and compromising locoregional factors. In 1995, the NEJM Fuchs published the results of gastrectomy in patients with compromised serosa and/or lymph nodes. These were not very encouraging as only a five-year survival period was achieved for only 29% and locoregional failures [1] was observed in 40%. On the other hand, García and collaborators reviewed 423 cases treated with surgery in a hospital in Santiago [2]. The overall survival (OS) rate of the group at five years was 33%. Patients with stage III gastric cancer had a survival rate of 19.6% to 38%, which compares favourably with the Memorial Sloan Kettering series published in 2000 [3]. In addition, a group from the University of Valparaíso published the results from 85 patients, who were operated on with R0 resection, resulting in a five-year survival rate of 20.7–33.3% in patients with nodal involvement [4]. Subsequently in the Hospital de la Universidad de Chile, Csendes and colleagues were able to carry out a prospective randomised study of D2 gastrectomy with splenectomy versus the same surgery without a splenectomy. There were no significant differences between the approaches, so they recommend not performing routine splenectomy. When looking at the survival rates of these patients who had very good quality surgery, we can see that the results in stages III range from 20–40% [5].

## Objectives

This review of scientific literature aims to expose data suggesting that the best adjuvant therapy for patients with advanced non-metastatic gastric cancer is a combination of radiotherapy and concomitant chemotherapy.

## Description and analysis of the literature

The poor results obtained in the series of patients treated exclusively with surgery forced the issue of exploring adjuvant treatments. A collaborative group comprising the IRAM Clinic in Santiago, the Clínica Reñaca, and the Naval Hospital in Viña del Mar led by Dr. Baeza published a study with 52 patients with gastric cancer. They underwent complete resection (R0) and received adjuvant treatment with radiotherapy and concomitant chemotherapy (RTQT). All had nodal or serous involvement. The five-year survival rate was quite encouraging, reaching 54% in a group of patients in which 86.5% had nodal involvement. The protocol was not harmless, but it is noteworthy that 100% of the patients completed it and 73% of them did so within the established time frame [6]. In 2008, Garrido and colleagues reviewed all the patients treated at the Catholic University with D2 gastrectomy plus RTQT. They compared their results with a historical cohort from their hospital with the same surgery, but without adjuvant treatment. Patients who received RTQT had a better survival rate and no patient discontinued the treatment. They considered that the toxicity of the adjuvant regime was low [7].

The first randomised trial to demonstrate a global survival benefit for an adjuvant treatment in gastric cancer was that of MacDonald published in 2001 [8]. The RTQT decreased locoregional recurrences and this was manifested in a 9% increase in OS at three years (from 41–50%). After more than ten years of follow-up, this study updated its data, and the benefit in OS with the highest follow-up persisted without the appearance of late aggregate toxicity. The criticism of this work is that the majority of these patients had resections considered insufficient by the standards of other regions of the world. Therefore, it is questioned if a more radical surgery (D2) could make the use of RT unnecessary. Although D2 resection is recognised as a standard practice in patients with gastric cancer, this intervention has failed to demonstrate a benefit in OS versus less radical dissection (D1). This has been studied in several randomised studies. When grouped in a meta-analysis, it can be seen that the radical intervention is not harmless as it produces a significant increase in hospital stay, postoperative complications, mortality, anastomotic leak, and re-interventions [9].

On the other hand, adjuvant therapy with chemotherapy alone has been evaluated i.e. taking the radiotherapy away from the treatment. The MAGIC study evaluates the use of chemotherapy for six cycles, three preoperative, and three postoperative versus only surgery. It was observed that adding chemotherapy gives a benefit in OS to patients. This despite the fact that the complete protocol was received only by 42% of the experimental arm. It appears that after three cycles of chemotherapy and a gastrectomy, it is difficult to receive more chemotherapy [10]. Another study assessing adjuvance with chemotherapy is the CLASSIC study which is a collaboration between centres in South Korea, China, and Taiwan and published its first results in 2012. In this study, more than a thousand patients were randomly assigned a D2 gastrectomy versus the same surgery and six months of adjuvant chemotherapy with capecitabine and oxaliplatin. There was an OS benefit with adjuvant treatment but only 66.5% of the patients were able to complete it, and a grade 3-4 toxicity was obtained in 56% of them. Once again it is verified that adding treatments to the surgery improves its results, but in this case at a high price because of toxicity [11]. The next question is how this treatment is compared with adjuvant RTQT.

The only randomised study comparing chemotherapy versus RTQT as an adjuvant treatment in patients with gastric cancer, i.e. who have had a gastrectomy and D2 dissection, is the ARTIST study. In order to gain all we can learn from this work, we must analyse the three publications of the latter [12, 13, 14]. The main objective of the study was to show a difference in disease-free survival (DFS) (not OS). It was not a large study as it only randomised 458 patients. The RTQT arm was better tolerated and could be completed in 81.7% of patients versus 75.4% of patients receiving adjuvant chemotherapy. The three-year DFS was higher for RTQT versus chemotherapy (78% versus 74%), but this did not achieve statistical significance even though the p-value is close (p = 0.0862). The main reason for not achieving a significant p-value was that the study included many patients without lymph node involvement, in whom adequate adjuvant chemotherapy is enough. In fact, when analysing the DFS of all patients with node involvement, this was better for those who received RTQT with a significant p-value (p = 0.0365). Added to this, the adjuvant chemotherapy arm had 3% more N0 patients, and hence had a better prognosis. The third publication of this study is the most interesting, as it makes a more in-depth analysis of the results. In patients with nodal involvement, locoregional recurrence was 14.5% for the adjuvant chemotherapy arm versus only 6.4% in the RTQT arm (p = 0.009). We should note that this is in the regions with the best or most radical surgery in the world. Another point to emphasise is the protocol of the radiotherapy that was used. The technique used was anterior-posterior fields. The one used here was a 2D radiotherapy which was extremely old, only slightly homogeneous, inaccurate, and poorly shaped for treatment of targets which were very irregularly shaped (splenic hilum, hepatic hilum, anastomosis, celiac trunk, etc.) and with many healthy organs to avoid irradiating (small intestine, large intestine, kidneys, liver, etc.). They did not use simulation with computed tomography or 3D conformational dosimetry, both standard in radiotherapy throughout Chile for at least a decade. It is noteworthy that even with poor radiation therapy, there has been a benefit in some patients. There is a study from the Princess Margaret Cancer Centre in Toronto which shows that their specialists prefered more tailored treatments such as intensity modulated radiotherapy (IMRT) for this kind of pathology. This is because with this technique the coverage of the region to be irradiated is far better. The region receives the dosage it is meant to and not less, and the doses given to the healthy organs are smaller [15]. It should be noted that this technique is also available in most radiotherapy centres in Chile.

**Figure 1.**
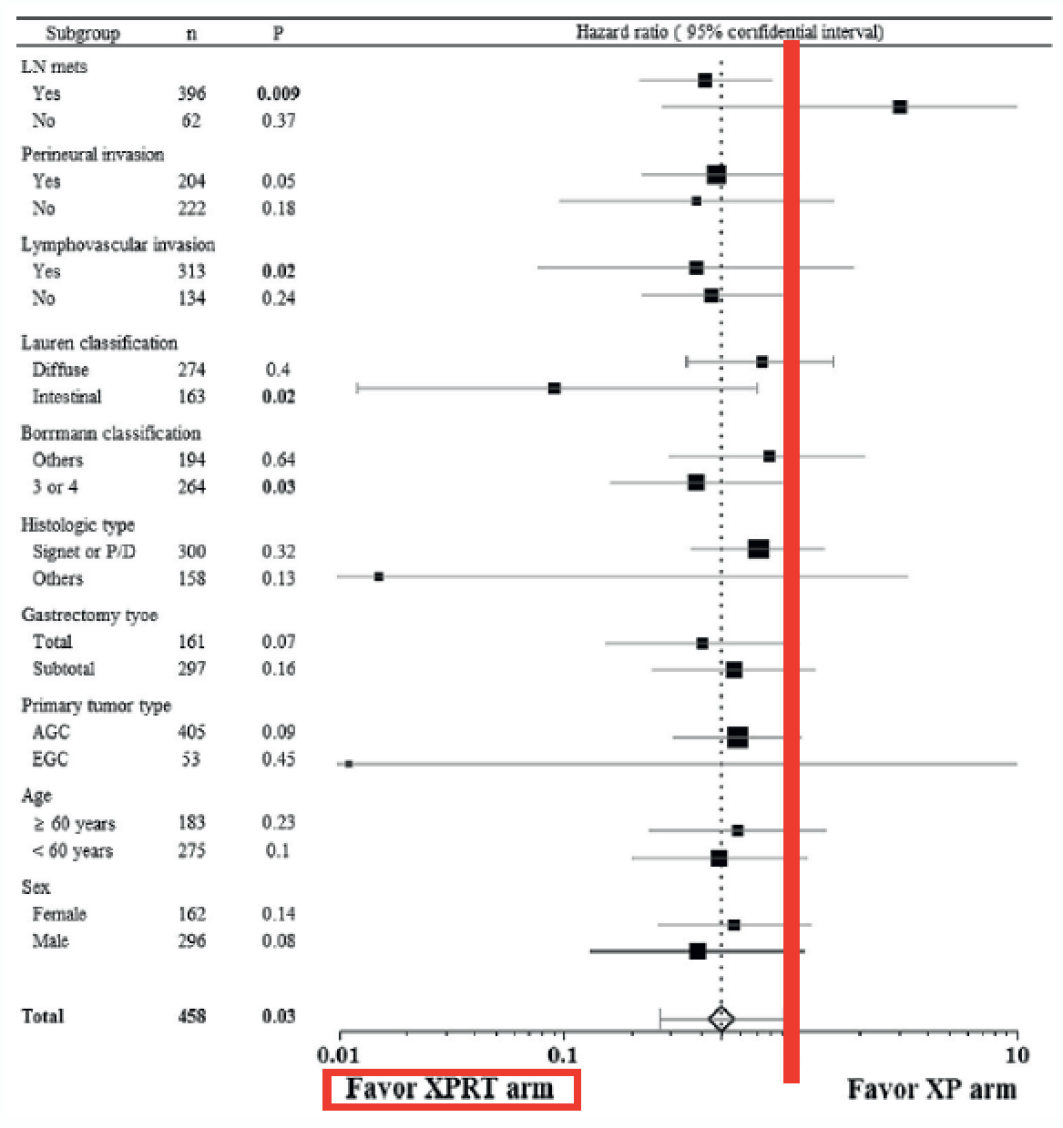
ARTIST trial subgroup analysis for disease-free survival. To the left of the red line, results favor RTQT. To the right of the red line, results favor chemotherapy.

In addition, the ARTIST study highlights the Forest Plot that brings forth the results in the different subgroups. All subgroups show the benefits of RTQT, except for patients without lymph node involvement (see Figure 1).

At the American Society of Clinical Oncology (ASCO) 2016 annual meeting, the initial results of the CRITICS study, which has not yet been published, were presented. This randomised study compares neoadjuvant chemotherapy followed by surgery and subsequently more chemotherapy versus the same neoadjuvant treatment but RTQT after surgery. There were no differences between the arms but only 52% of the patients [16] randomised to RTQT received it. Whereas in the MAGIC study, after neoadjuvant treatment and surgery, a large number of patients did not tolerate further treatment. We are awaiting the publication of this study.

## Future publications

There are two very interesting studies which are recruiting patients. One is the ARTIST-II (NCT01761461) study which randomises patients with D2 resection and nodal involvement into three-arms, two for chemotherapy and one for RTQT. By 2014, there were already 118 randomised patients. Another very interesting study is that of TOPGEAR (NCT01924819), which for the first time evaluates neoadjuvant RTQT treatment in gastric cancer versus neoadjuvant chemotherapy alone. By 2015, there were 170 randomised patients.

## Conclusion

D2 gastrectomy plus adjuvant chemotherapy is not sufficient for locally advanced gastric cancer. The only comparison of this strategy versus adjuvant RTQT (ARTIST) shows that with surgery and chemotherapy, locoregional recurrence is 14.5% versus only 6.4% when RTQT is carried out (p = 0.009). In addition, the RTQT scheme has less toxicity and can be completed by a greater proportion of patients. We must also consider that present radiotherapy worldwide has improved greatly with respect to radiotherapy used at the time of the randomised trials which even then had proved to be beneficial for this pathology.
